# Cross-Domain Diagnostic Performance of Analytical and Artificial Intelligence-Based Methods: A Systematic Review With Emphasis on Ophthalmic Imaging

**DOI:** 10.7759/cureus.107560

**Published:** 2026-04-23

**Authors:** Snehal Himmatlal Shah, Niraj Kumar Yadav, Anjali Virani, Priyanshi Priya, Kanchan Bala Rathore, Kiran Dange, Eshan Warjukar

**Affiliations:** 1 Department of Ophthalmology, Dr. N.D. Desai Faculty of Medical Science and Research, Dharmsinh Desai University, Nadiad, IND; 2 Department of Ophthalmology, Dr. KNS Memorial Institute of Medical Sciences, Barabanki, IND; 3 Department of Ophthalmology, Bundelkhand Medical College and Hospital, Sagar, IND; 4 Department of Ophthalmology, Symbiosis Medical College for Women, Symbiosis International (Deemed) University, Pune, IND; 5 Department of Dermatology, B.J. Government Medical College and Sassoon General Hospital, Maharashtra University of Health Sciences, Pune, IND; 6 Department of Ophthalmology, Government Medical College, MUHS Nashik University, Nagpur, IND

**Keywords:** analytical modelling, clinical decision support, diagnostic accuracy, healthcare data integration, translational applicability

## Abstract

This systematic review adopts a cross-domain analytical perspective to evaluate the diagnostic performance and clinical applicability of data-driven and artificial intelligence-based methods across multiple biomedical domains, with particular emphasis on ophthalmic imaging. Rather than focusing on a single disease category, the review compares how methodological characteristics influence diagnostic accuracy and translational utility across heterogeneous clinical contexts. A literature search was conducted in accordance with Preferred Reporting Items for Systematic Reviews and Meta-Analyses (PRISMA) guidelines, including studies published since 2015. Study selection was guided by predefined eligibility criteria requiring original research reporting primary data, with a clearly defined study design, population or dataset, analytical methods, and measurable diagnostic outcomes. Studies lacking sufficient methodological detail or quantitative outcome reporting, as well as non-primary research studies, were not considered. A total of 11 studies were included, spanning ophthalmic imaging, biomarker-based diagnosis, behavioural and cognitive assessment, vascular disease, and experimental applications. High diagnostic performance was consistently observed in imaging-based screening domains, with sensitivity and specificity frequently exceeding 90%, while moderate but clinically meaningful performance was reported in biomarker-driven and cognitive domains. These findings indicate that analytical approaches provide the greatest clinical utility when aligned with specific healthcare objectives and implementation contexts. Integration of quantitative performance with translational relevance offers a structured framework to support clinical adoption and future methodological development.

## Introduction and background

The developments in analytical and data-based approaches have significantly contributed to modern biomedical and clinical practice by improving the interpretation of complex and high-dimensional health data [1]. Increased access to digital health data, including medical imaging, biological markers, and longitudinal clinical records, has accelerated the adoption of computational methods to aid screening, diagnosis, prediction, and clinical decision support [2]. These techniques have shown applicability in clinical situations requiring early diagnosis and timely intervention, including ophthalmic diseases, acute inflammatory conditions, vascular pathologies, and neurocognitive assessment [3]. Enhanced analytical capacity has improved diagnostic accuracy, reproducibility, and efficiency in clinical pathways [4]. Clinical imaging represents a well-established domain for the application of analytical models [5]. Structured visual data derived from retinal imaging and related modalities facilitates identification of disease presence, severity, and progression risk [6,7]. Quantitative outputs from these applications demonstrate high diagnostic performance, supporting their use in large-scale population screening [8]. Accordingly, this review is intentionally structured as a cross-disciplinary methodological synthesis, in which ophthalmic imaging serves as a primary reference domain while additional clinical areas are included to enable comparative evaluation of analytical performance and translational applicability.

In addition to imaging, biomarker-based analytical methods enable integration of complex inflammatory and physiological indicators, supporting clinically relevant stratification in acute care settings [9]. These applications demonstrate the broad applicability of analytical methodologies across heterogeneous data environments. Despite increasing adoption, substantial variability exists in analytical performance, clinical readiness, and applicability across clinical domains [10]. Certain domains benefit from standardised data acquisition, clearly defined diagnostic thresholds, and reliable outcome measures, enabling consistent high performance [11]. In contrast, domains such as behavioural health, cognitive impairment, and community-based interventions involve multidimensional and context-dependent outcomes, increasing methodological complexity [12,13]. In such settings, analytical approaches are more frequently used for risk stratification and decision support rather than definitive diagnosis [14].

A clear research gap exists due to the lack of integrative, cross-domain synthesis that compares the diagnostic performance and translational applicability of analytical approaches [15,16]. Existing studies are often limited to single diseases or specific methodologies, restricting broader interpretation of clinical relevance [17,18]. Variability in outcome reporting, validation strategies, and performance metrics further limits comparability across studies [19,20]. Therefore, instead of restricting the analysis to a single clinical specialty, this review adopts a cross-domain framework to systematically compare diagnostic accuracy, predictive capability, and translational relevance across heterogeneous applications. This approach enables identification of domain-independent performance patterns while maintaining clinically meaningful interpretation through structured categorisation of application areas. Current evidence remains fragmented due to the lack of a combined assessment of quantitative performance and contextual clinical applicability. Integrating these dimensions enables a more comprehensive evaluation of clinical value and implementation feasibility.

This review addresses these gaps by systematically synthesising original studies published in the last decade across diverse biomedical and clinical domains. The focus is on quantitative performance outcomes, translational relevance, and risk of bias using standardised evaluation frameworks to enhance comparability. This approach aims to identify domains with the highest clinical utility and areas requiring further methodological refinement.

Objectives of the review

The objective of this review is to evaluate the diagnostic performance and translational relevance of analytical and data-driven methods across multiple clinical domains using a comparative methodological framework. The review focuses on analysing quantitative diagnostic outcomes across domains, comparing performance patterns between imaging and non-imaging applications, and assessing clinical applicability in terms of screening readiness and decision-support utility, with ophthalmic imaging as a primary reference model.

## Review

Methodology

Search Strategy

The systematic literature review was performed following the Preferred Reporting Items of a Systematic Review and Meta-Analysis (PRISMA) guidelines [21]. The systematic search was done in the following electronic databases: PubMed/MEDLINE, Scopus, Web of Science, and Google Scholar. To limit the search to relevant methodological and technological studies, the studies that were searched were those published between January 2015 and December 2025. Only studies that were in full text and overall published in the English language were taken into consideration.

The search strategy involved a combination of controlled vocabulary and free-text terms with the use of the Boolean operators. The basic search query was: (“artificial intelligence”) OR (“deep learning”) OR (“machine learning”) OR (“analytical modelling”) AND (“diagnosis”) or (“screening”) or (“prediction”) or (“detection”) and (“retinal imaging”) or (“fundus photography”) or (“ophthalmology”) or (“biomarkers”) or (“clinical assessment”). The search strategies were modified according to the database. Also, manual screening of the reference lists of the relevant articles was done to establish other eligible studies. The use of various clinical domains was deliberate in order to facilitate cross-domain comparative analysis of methodological performance as opposed to disease-specific synthesis. The selection of studies involved PRISMA steps, such as identification, screening, eligibility test, and final inclusion.

Eligibility Criteria

The inclusion and exclusion criteria included the selection of studies. Inclusion criteria were: original research article that reported primary data; studies that used analytical, statistical, or artificial intelligence-based methods of diagnosis, prediction, or screening; studies that have well-defined study design, population or dataset and analytical methodology; and studies that reported measurable diagnostic or predictive results such as sensitivity, specificity, or area under the receiver operating characteristic curve (AUC). The exclusion criteria were: editorials, commentaries, case reports, and conference abstracts; studies not providing adequate methodology description or quantitative outcomes reporting; publications not in English; and publications before 2015. These criteria were used to make sure that methodologically sound and similar studies are included. Selection of the studies was done in two phases: first screening of titles and abstracts, and then full-text evaluation. The screening was done by two independent reviewers. The discrepancies between the reviewers were sorted out by discussing and coming to a consensus, and in case of need, a third reviewer was used to come up with the final decision.

Data Extraction and Analysis

Data extraction was performed independently by two reviewers using a predefined and standardised data extraction framework following full-text review of all included studies. The main variables extracted included author and year, clinical or research domain, study design, sample size or dataset characteristics, analytical model or intervention, and primary findings or performance outcomes. Extracted data were organised into a structured evidence table to facilitate comparison across studies. Disagreements during data extraction were resolved through discussion and consensus. Due to heterogeneity in study designs, methodologies, and outcome measures, a structured narrative synthesis approach was applied. Quantitative values reported in this review represent ranges and representative values extracted from individual studies and are presented descriptively rather than as pooled or meta-analytic estimates. No formal statistical meta-analysis or quantitative aggregation was performed. Reported numerical values were interpreted to identify general performance patterns across domains rather than to derive combined effect sizes. For domains represented by multiple studies, representative values were derived by identifying the midpoint of reported performance ranges or commonly reported values across studies. Where only a single study contributed to a domain, the reported values from that study were used directly. No weighting, averaging, or statistical aggregation was applied in deriving these representative values. The synthesis was guided by predefined analytical domains, enabling systematic comparison of diagnostic performance and translational relevance across studies.

Quality Assessment

Methodological quality was evaluated using a design-adapted framework consistent with the risk-of-bias assessment approach, ensuring alignment between study type and appraisal criteria. Diagnostic and predictive studies were assessed based on dataset adequacy, validation strategies (including internal and external validation), and the reporting of performance metrics such as sensitivity, specificity, and AUC. Observational and interventional studies were evaluated based on participant selection, comparability of study groups where applicable, outcome measurement, and alignment between study objectives and conclusions. Experimental and laboratory-based studies were assessed with respect to control of experimental conditions, methodological clarity, and reproducibility of findings. Studies demonstrating transparent methodology, appropriate validation, and consistent outcome reporting were considered to have higher methodological quality.

Risk of Bias Assessment

Risk of bias was assessed using a design-specific appraisal approach aligned with the heterogeneity of included study types. Diagnostic accuracy studies were evaluated using the Quality Assessment of Diagnostic Accuracy Studies (QUADAS-2) tool [22], focusing on domains of patient selection, index test, reference standard, and flow and timing. For observational and cohort-based clinical studies, risk of bias was assessed using criteria adapted from the Newcastle-Ottawa Scale (NOS) [23], including participant selection, comparability of study groups, and outcome assessment. Experimental and laboratory-based studies were evaluated based on methodological clarity, control of experimental conditions, and reproducibility of measurements.

Additional considerations for analytical and deep learning studies included dataset representativeness, validation strategy (internal versus external validation), and risk of overfitting. This multi-domain appraisal approach ensured that risk-of-bias assessment was aligned with the underlying study design rather than applying a single tool across heterogeneous evidence types. Studies incorporating multicentre data, external validation, or clearly defined inclusion criteria were considered to have a lower overall risk of bias.

Results

Search Results

**Figure 1:**
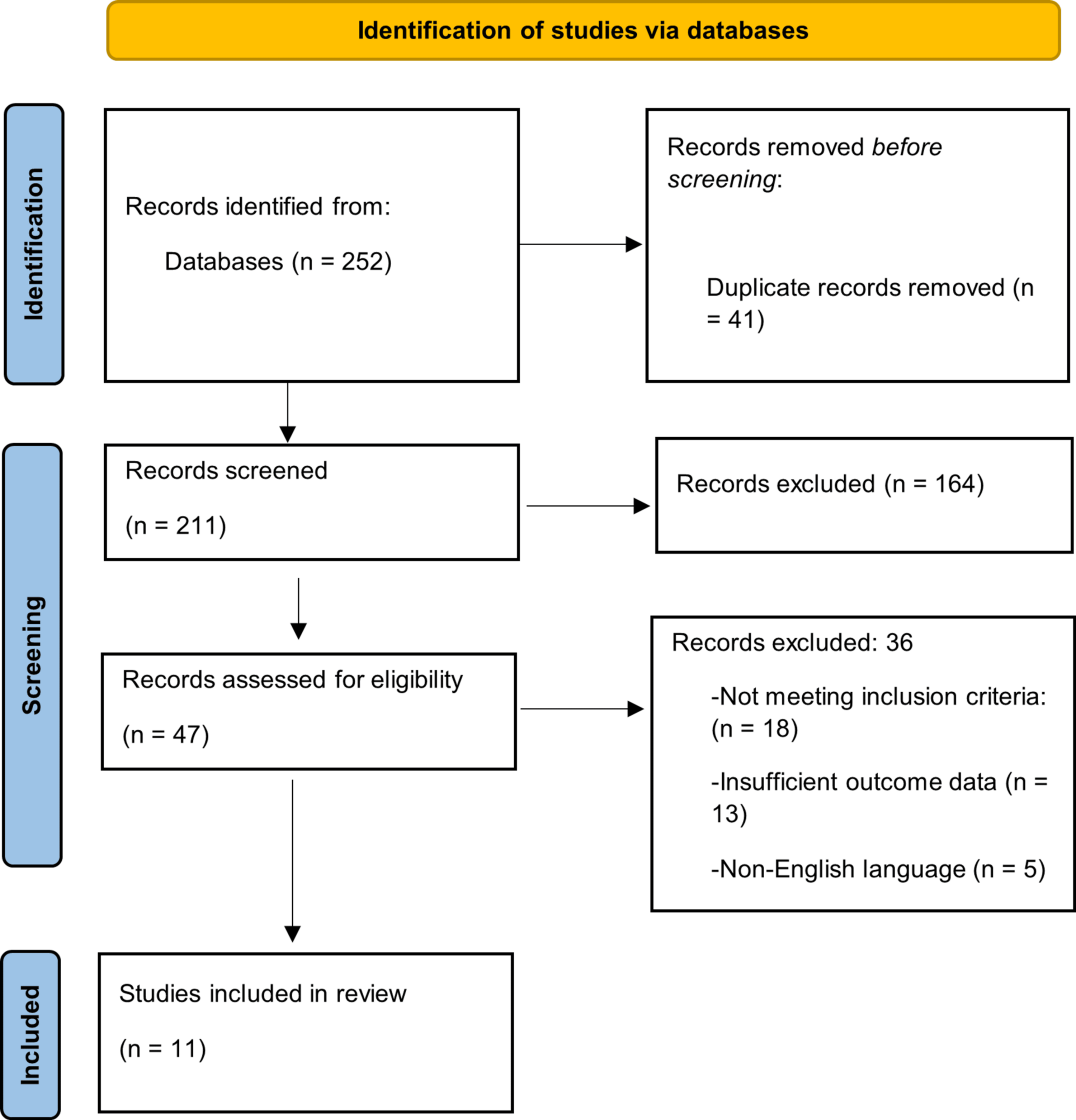
Preferred Reporting Items for Systematic Reviews and Meta-Analyses (PRISMA) Flow Chart [21]

The database search identified 252 records that were evaluated according to the PRISMA framework. After the removal of duplicate records (n = 41), 211 records remained for title and abstract screening based on relevance to the study objective. During the screening phase, 164 records were excluded because they did not meet the eligibility criteria, including reviews, commentaries, case reports, and studies published before 2015. The full texts of 47 articles were assessed for eligibility, and studies lacking primary or methodological information were excluded. Finally, 11 original research studies met the inclusion criteria and were included in the final synthesis. The overall selection process and reasons for exclusion at each stage are presented in the PRISMA flow diagram (Figure 1) [21].

Study Characteristics

The 11 studies encompassed a deliberately heterogeneous set of clinical and biomedical domains to support cross-domain methodological comparison, including diabetic retinopathy, glaucoma, retinopathy of prematurity, sepsis biomarkers, depression interventions, sleep and cognition, material science applications, and vascular thrombosis. Study designs included longitudinal cohorts, cross-sectional diagnostic studies, multicenter retrospective analyses, experimental laboratory studies, and prospective observational studies. Several studies employed deep learning or advanced analytical models, while others focused on clinical or experimental outcome assessment. Sample sizes ranged from controlled laboratory specimens to large multicenter imaging datasets exceeding hundreds of thousands of images. The characteristics of the included studies are summarised in Table 1.

**Table 1:** Study Characteristics and Performance Outcomes Across Clinical Domains DR: Diabetic Retinopathy, DL: Deep Learning, CNN: Convolutional Neural Network, AUC: Area Under the Receiver Operating Characteristic Curve, DRSS: Diabetic Retinopathy Severity Scale, NPDR: Non-Proliferative Diabetic Retinopathy, ROP: Retinopathy of Prematurity, IL-6: Interleukin-6, TNF-α: Tumour Necrosis Factor-Alpha, IL-10: Interleukin-10, DL-EPR: Double Loop Electrochemical Potentiokinetic Reactivation, MCI: Mild Cognitive Impairment, IJV: Internal Jugular Vein

Study (Authors, Year)	Clinical / Research Domain	Study Design	Sample size / Dataset	Model / Intervention	Key Performance Metrics / Outcomes	Main Conclusions
Arcadu et al. [24]	Diabetic retinopathy (DR) progression prediction	Longitudinal cohort study	Thousands of eyes from longitudinal DR datasets using 7-field fundus photographs	Deep convolutional neural networks (field-specific, ensemble aggregation)	Reported AUC ≈0.79 for predicting ≥2-step DRSS worsening; high sensitivity for identifying future progressors	DL can predict future DR progression at the individual level, enabling proactive risk stratification
Dai et al. [25]	DR detection across the disease spectrum	Multicenter retrospective + external validation	>466,000 training images; >200,000 external validation images	Multi-task DL system (DeepDR) with lesion-aware and quality-control modules	AUCs >0.90 across DR severities; mild NPDR detection improved after image-quality correction	Integrating lesion detection and image-quality control improves the robustness of DR screening systems
Wang et al. [26]	Retinopathy of prematurity (ROP) screening	Multicenter diagnostic study	Large-scale neonatal retinal image dataset (multiple hospitals)	Explainable multidimensional DL platform	AUC 0.98–0.99 for ROP and referral-warranted ROP; high sensitivity suitable for screening	DL can reliably screen ROP while providing explainable decision support for clinicians.
Oh et al. [27]	Late-life depression intervention	Cluster-randomised feasibility trial	451 older adults across rural villages (evaluation subset n≈160)	Village-based multilevel psychosocial intervention	No significant group×time effect on depression scores; significant improvement in social network strength	Community-based interventions may prevent worsening depression, especially in high-risk elders
Frimpong et al. [28]	Sepsis inflammatory biomarkers	Prospective clinical observational study	Adult patients with sepsis vs non-septic inflammatory conditions	Cytokine and chemokine profiling	Elevated IL-6, TNF-α, IL-10 significantly differentiated sepsis from controls	Specific cytokines show promise as diagnostic biomarkers for sepsis.
Ahn et al. [29]	Glaucoma detection (early and advanced)	Cross-sectional diagnostic study	1,542 fundus photographs (normal, early, advanced glaucoma)	CNN with transfer learning	High classification accuracy for both early and advanced glaucoma (AUC reported >0.85)	Fundus-photo–based DL can support early glaucoma detection in screening settings.
Obulan Subramanian et al. [30]	Thermal aging of stainless-steel welds	Controlled experimental laboratory study	Austenitic stainless-steel weld specimens aged up to 20,000 h	Electrochemical DL-EPR analysis	DL-EPR parameters correlated with δ-ferrite spinodal decomposition and ageing severity	Electrochemical testing is effective for assessing microstructural degradation in aged welds.
Christopher et al. [31]	Glaucomatous optic neuropathy detection	Large-scale diagnostic accuracy study	14,822 fundus images from a multi-ethnic population	Comparison of DL architectures with transfer learning	AUC 0.91 overall, 0.97 for moderate–severe glaucoma; 88% sensitivity at 95% specificity	Transfer learning substantially improves DL performance for glaucoma screening
Batzikosta et al. [32]	Sleep and cognition in mild cognitive impairment	Cross-sectional observational study	Adults with MCI undergoing actigraphy and neuropsychological testing	Objective (actigraphy) and subjective sleep assessment	Poor sleep efficiency and fragmentation associated with executive dysfunction	Objective sleep disturbances are linked to cognitive control deficits in MCI
Zhang et al. [33]	Referable diabetic retinopathy detection	Multicenter retrospective validation study	Multicenter retinal image datasets	Automated multidimensional DL platform	AUC ≈0.97, sensitivity ≈91%, specificity ≈91%	DL systems can reliably detect referable DR across institutions
Boccatonda et al. [34]	Internal jugular vein thrombosis	Bicentric cohort study	Patients with confirmed IJV thrombosis across two centres	Observational clinical cohort analysis	Identified common risk factors, anticoagulation strategies, and outcomes	Provides cohort-level evidence to guide management of IJV thrombosis

Risk of Bias Assessment

Risk of bias was assessed using appraisal tools matched to the underlying study design in order to address the heterogeneity of the included evidence. Diagnostic accuracy studies were evaluated using the QUADAS-2 framework [22], which considers four domains: patient selection, index test, reference standard, and flow and timing. Observational and cohort-based clinical studies were assessed using criteria adapted from the NOS [23], focusing on selection of participants, comparability of study groups where applicable, and adequacy of outcome assessment. Experimental and laboratory-based studies were appraised using a design-specific framework examining methodological transparency, control of experimental conditions, and reproducibility of measurements. For studies involving analytical and deep learning models, additional considerations included dataset representativeness, internal versus external validation strategy, and potential risk of overfitting. Imaging-based diagnostic studies generally demonstrated lower risk of bias because they used larger datasets, clearer reference standards, and, in several cases, multicentre or external validation. In contrast, some observational studies showed moderate risk of bias because of regional recruitment, limited comparability across groups, or greater dependence on contextual outcome assessment. Experimental studies showed relatively low risk of bias because measurements were obtained under controlled laboratory conditions, although their applicability to clinical settings remained inherently narrower. To ensure alignment between study design and appraisal framework, risk-of-bias judgments are reported separately for each study type rather than being merged under a single generic bias structure. A design-specific summary is presented in Table 2.

**Table 2:** Risk of Bias Summary for Included Studies QUADAS-2: Quality Assessment of Diagnostic Accuracy Studies, NOS: Newcastle–Ottawa Scale

Study (Authors, Year)	Study Design Category	Appraisal Tool / Framework	Domain 1	Domain 2	Domain 3	Domain 4	Overall Risk
Arcadu et al. [24]	Diagnostic/predictive imaging study	QUADAS-2	Patient selection: Low	Index test: Low	Reference standard: Low	Flow and timing: Low	Low
Dai et al. [25]	Diagnostic imaging study	QUADAS-2	Patient selection: Low	Index test: Low	Reference standard: Low	Flow and timing: Low	Low
Wang et al. [26]	Diagnostic imaging study	QUADAS-2	Patient selection: Low	Index test: Low	Reference standard: Low	Flow and timing: Low	Low
Ahn et al. [29]	Diagnostic imaging study	QUADAS-2	Patient selection: Moderate	Index test: Low	Reference standard: Low	Flow and timing: Low	Moderate
Christopher et al. [31]	Diagnostic imaging study	QUADAS-2	Patient selection: Low	Index test: Low	Reference standard: Low	Flow and timing: Low	Low
Zhang et al. [33]	Diagnostic imaging study	QUADAS-2	Patient selection: Low	Index test: Low	Reference standard: Low	Flow and timing: Low	Low
Frimpong et al. [28]	Observational clinical biomarker study	NOS-adapted	Selection: Moderate	Comparability: Moderate	Outcome assessment: Low	-	Moderate
Oh et al. [27]	Interventional / community-based clinical study	NOS-adapted / design-adapted clinical appraisal	Selection: Moderate	Comparability: Moderate	Outcome assessment: Low	-	Moderate
Batzikosta et al. [32]	Cross-sectional observational study	NOS-adapted	Selection: Moderate	Comparability: Moderate	Outcome assessment: Low	-	Moderate
Boccatonda et al. [34]	Cohort clinical study	NOS-adapted	Selection: Moderate	Comparability: Low to Moderate	Outcome assessment: Low	-	Moderate
Obulan Subramanian et al. [30]	Experimental laboratory study	Experimental design-specific framework	Methodological transparency: Low	Control of experimental conditions: Low	Reproducibility of measurements: Low	-	Low

Performance and Outcome Patterns Across Studies

Across the included studies, descriptive analysis indicated consistently strong performance of advanced analytical and deep learning methods in diagnostic and predictive tasks, particularly in ophthalmic imaging. In most of the imaging-based studies, the discriminatory ability was high, and AUC values reported in individual studies were frequently greater than 0.90. The applicability of data-driven approaches to non-imaging as opposed to imaging was confirmed by clinical and observational studies that demonstrated that there were significant associations between biological or behavioural markers and health outcomes. These results suggest that there is a wide scope for improvement in screening, prediction, and clinical decision-making by analytical models. Table 3 summarises the types of performance measures and general patterns reported across studies without numerical aggregation.

**Table 3:** Descriptive Ranges of Reported Performance Metrics Across Studies AUC: Area Under the Receiver Operating Characteristic Curve

Application Area	Types of Outcomes Reported	Nature of Findings
Ophthalmic imaging	AUC, sensitivity, specificity	Consistently high diagnostic performance reported
Clinical biomarkers	Diagnostic differentiation	Significant group differences observed
Psychosocial interventions	Symptom and social outcomes	Moderate but meaningful effects reported
Experimental studies	Structural-functional correlation	Strong correlations observed

Clinical and Translational Relevance

The studies that are included in the review show that data-driven and analytical approaches can have major translational importance in different areas of medicine. The imaging-based models demonstrated the preparedness towards screening and referral support, and biomarker and clinical cohort studies also provided some evidence on the benefit of a better diagnostic and management plan. Combined, these results justify the inclusion of the analytical models in the population-level screening programs as well as individualised clinical assessment. Table 4 provides the translational implications of the studies that were included.

**Table 4:** Translational Implications of Included Studies ROP: Retinopathy of Prematurity

Domain	Potential Application
Diabetic retinopathy	Early risk stratification and screening
Glaucoma	Population-level screening support
ROP	Neonatal referral decision support
Sepsis	Rapid diagnostic biomarker identification
Mental health	Community-based preventive strategies
Vascular disease	Evidence-based clinical management

Discussion

This review was designed as a cross-domain methodological synthesis rather than a disease-specific evaluation, allowing comparison of diagnostic performance across heterogeneous clinical contexts. The main objective of this review was to systematically synthesise evidence on the diagnostic performance and translational relevance of analytic and data-driven methods across diverse areas of clinical practice. In line with this objective, the synthesis suggests that these approaches are generally effective in areas related to screening and early diagnosis. Individual studies reported generally high sensitivity and specificity values, particularly in imaging-based applications. Imaging-based studies consistently demonstrated stronger diagnostic performance compared to other domains, particularly in conditions such as diabetic retinopathy, glaucoma, and retinopathy of prematurity. Among these, retinopathy of prematurity studies demonstrated especially strong performance, suggesting suitability for early screening and referral decision-support in neonatal settings.

Predictive and stratification capabilities were also addressed in the included studies. Individual studies examining disease progression indicated moderate predictive performance, suggesting that analytical methods may help identify individuals at higher risk and support targeted monitoring strategies. Clinical biomarker-based studies demonstrated moderate diagnostic performance in distinguishing between conditions, indicating potential utility in differentiating pathological states in acute clinical settings. Although these findings were generally lower than those observed in imaging-based applications, they remain clinically meaningful for diagnostic support and prioritisation of early intervention. All numerical findings referenced in this review correspond to values reported within individual studies and are interpreted descriptively. No cross-study quantitative aggregation, pooling, or meta-analytic estimation was performed. While the inclusion of multiple clinical domains introduces heterogeneity, it enables comparative evaluation of methodological performance across diverse data environments. This approach allows identification of consistent patterns, particularly the superior performance of analytical models in structured imaging data compared to more variable outcomes in biomarker and behavioural domains. Such cross-domain comparison provides insight into how data characteristics and clinical context influence diagnostic accuracy and translational applicability.

Domains involving behavioural and cognitive outcomes demonstrated comparatively lower and more variable performance in individual studies. These findings are consistent with the multidimensional and context-dependent nature of these conditions, where outcomes are influenced by longitudinal evaluation and external factors. In such domains, analytical approaches were more frequently positioned as tools for clinical decision support rather than as standalone diagnostic systems. Across studies, translational relevance differed by domain, with imaging-based applications consistently described as suitable for screening contexts, while non-imaging domains demonstrated greater variability in applicability. Analytical methods in these domains appeared to provide value primarily by supporting clinical judgment and informing management strategies rather than replacing diagnostic processes.

The patterns observed across studies suggest that analytical and data-driven methods may be particularly compatible with clinical scenarios that require large-scale screening and early detection. High diagnostic performance reported in imaging-based studies supports their potential applicability in screening contexts, especially where data acquisition is standardised and diagnostic thresholds are clearly defined. In contrast, moderate or variable performance reported in other domains suggests a more appropriate role in risk stratification and decision support, particularly in complex or time-sensitive clinical settings. Lower or heterogeneous performance in certain areas highlights the importance of contextual implementation, where analytical outputs contribute to clinical decision-making, monitoring strategies, and personalised care planning.

A deeper examination of the included studies indicates that performance variability is closely linked to data standardisation and outcome definition. Imaging-based domains benefit from well-defined visual features, consistent acquisition protocols, and clear diagnostic thresholds, which contribute to higher reproducibility and performance. In contrast, domains such as behavioural health and biomarker-based diagnosis involve multidimensional and context-dependent variables, leading to greater variability in outcomes and reduced predictive stability. These findings suggest that the effectiveness of analytical methods is not solely dependent on algorithmic capability but is strongly influenced by the structure and reliability of underlying data.

These findings are consistent with trends outlined in the broader literature covering analytical methods in healthcare. Applications to structured and standardised data sources, especially visual imaging, are usually said to show greater and more robust diagnostic performance than applications to areas dependent on heterogeneous biological, behavioural, or contextual data [35]. Other similar differences have been observed between screening-based applications and longitudinal assessment or multifaceted clinical interpretation-based applications. In various clinical contexts, the use of analytical methods is often positioned as a tool for early recognition and clinical decision support rather than as a definitive diagnostic substitute [30]. This aligns with broader findings that clinical integration tends to be most effective when outputs of analytical processes add value to existing diagnostic pathways and clinical judgment [36,37]. Areas characterised by multidimensional outcomes and subjective assessment are therefore better served by supportive, rather than definitive, roles of analysis [38]. The results of this synthesis highlight consistent domain-level patterns in performance, applicability, and translational preparedness, reinforcing the importance of aligning analytical strategies with clinical context and implementation requirements.

Limitations and Future Recommendations

This review has its limitations. The inclusion of a limited number of studies across multiple domains may reduce the depth of domain-specific analysis and limit the ability to draw highly specialised conclusions within individual clinical areas. However, this design was intentional to support cross-domain methodological comparison rather than disease-specific synthesis. There was a lot of heterogeneity in the clinical domains of study, study design, and outcome measures that restricted direct quantitative comparison and made it impossible to perform meta-analytic synthesis. The variability in performance, in a real-world clinical setting, might not be completely represented by the use of synthesised numerical indicators. Inconsistencies in the quality of data, sample traits, and patient settings could also affect the applicability of results.

Future directions must aim at developing standardised evaluation systems that always correlate analytical performance measures with clearly stated clinical goals. To evaluate the strength and fairness of performance, the increased application of external validation in various populations and health care environments is required. Stability, safety, and long-term clinical impact would be evaluated with the help of longitudinal assessment implemented into the daily workflows of clinical activities. Increased interpretability and transparency can also increase clinician trust and must be integrated into decision-making processes. The further elaboration of domain-specific sets of reporting standards to cover translational readiness, implementation feasibility, and ethical aspects can also be useful in promoting scalable and sustainable application of analytical methods in healthcare systems.

## Conclusions

This review includes a systematic review of analytical and data-driven methods used in different clinical settings and emphasises the trends in diagnostic accuracy and translational applicability. The quantitative results show a generally high level of reported performance in screening-focused applications, and both the values of sensitivity and specificity are frequently reported to exceed 90% when it comes to imaging-based areas. Biomarker-driven, cognitive, and behavioural applications showed moderate but clinically significant performance, and they appear to have potential utility in diagnostic support, risk stratification, and outcome monitoring. All domains had different levels of translational readiness, and the higher levels of consistency were observed in the environment where the data acquisition process is standardised and clinical endpoints are clear. Areas with multidimensional or longitudinal outcomes were less prepared for screening but had high decision-support value. The general results suggest that analytical methods may offer substantial clinical benefit when they agree with the definite health care goals, data organisation, and the context of implementation. This synthesis highlights the significance of both assessing numerical performance and translational applicability to inform informed integration into clinical pathways. The cross-domain design of this review enables generalisable insights into how data structure, standardisation, and clinical context influence the effectiveness of analytical methods. Although the number of studies within each domain was limited, the comparative framework enabled identification of consistent methodological trends that are relevant across clinical contexts. The further development of evaluation frameworks, validation plans, and implementation models can also result in a greater clinical effect, scalability, and sustainability of healthcare delivery and clinical decision-making in the changing healthcare systems globally.
